# Improving Weight Bias Awareness Among Providers in the Sexual and Reproductive Health care Setting

**DOI:** 10.1089/heq.2024.0096

**Published:** 2024-07-01

**Authors:** Monica Skoko Rodriguez, Julie A. Thompson, Brigit Carter, Robin Wallace, Katie Riley, Ragan Johnson

**Affiliations:** ^1^ Duke University School of Nursing, Durham, North Carolina, USA.; ^2^ Planned Parenthood Federation of America, New York, New York, USA.; ^3^ Duke University, American Association of Colleges of Nursing, Washington, District of Columbia, USA.; ^4^ Planned Parenthood of Northern New England, Burlington, Vermont, USA.

**Keywords:** weight bias, stigma, health equity, reproductive health, healthcare providers

## Abstract

**Introduction:**

Provider bias against patients of higher weights can contribute to poor health outcomes and decreased quality of care and patient experience. Addressing weight stigma in sexual and reproductive health settings is important, as these encounters can often be patients’ only health care touchpoint. Health care providers must be educated about the harms of weight stigma, ways to recognize and confront their biases, and how to advocate for patients of all sizes.

**Methods:**

In this quality improvement project, Planned Parenthood health center providers participated in a three-part virtual workshop to improve provider weight bias awareness and understanding using the Health at Every Size framework. Providers completed a pre- and post-survey, as well as a 3-month follow-up survey to assess changes in bias awareness and confidence in applying weight-neutral principles in care interactions.

**Results:**

Analysis of pre- and post-survey results showed significant improvements in provider awareness of bias as well as changes in implicit bias scores and confidence providing weight-neutral care.

**Conclusion:**

Educating providers about weight contributes to equity of care for patients of higher weights. Formal education such as workshops have the potential to reduce the harms of weight stigma in health care as changing attitudes and confidence are a precursor to behavior change. Research is needed to assess ideal education modalities and whether receiving care from weight bias-prepared providers affects patient outcomes and experiences.

## Introduction

The Centers for Disease Control and Prevention report that 41.9% of American adults are considered obese by the body mass index (BMI) as of March 2020, an increase of over 10% from 2000.^1^ Fat patients (*when “fat” is used in this article, it refers to those categorized as overweight and obese in the BMI, as a growing body of work has championed the use of the word “fat” as a neutral descriptor of size.*^2,3^) are often subjected to weight bias by providers, potentially resulting in adverse physiological and psychological health outcomes and decreased quality of care.^4–6^ Given the high prevalence of Americans living at higher weights, and that weight discrimination may be linked to lower life expectancy,^7^ it is crucial for health care providers to be aware of their weight biases. Understanding and reducing bias can improve health outcomes, patient experience, and care utilization by fat patients,^8^ but very few providers are prepared to mitigate weight bias.^9^

Providers can increase their understanding of implicit and explicit biases that lead fat patients to avoid care. Weight shaming, incorrectly sized blood pressure cuffs, or lack of bariatric exam tables that include higher weights lead to inadequate care for fat patients.^4^ Providers may feel inadequately prepared to navigate care of fat patients in a respectful and evidence-based way^10^ and often spend less time and are less thorough with fat patients.^4^ Fat patients have reported being improperly assessed or simply told to lose weight, to later discover a serious condition or illness.^11^ Lower quality care due to bias is evident when fat patients are not given alternative care options to treatments that have lower efficacy in people of higher weights.^12^

There is a significant negative association between level of bias and quality of care.^13,14^ Common themes expressed by fat patients include providers conveying contempt, patronization, disrespect, and assuming all health issues are related to weight and conversely solvable through weight loss. Patients report that this stigma leads to mistrust of health care workers and providers, avoidance of care, and the phenomenon of “doctor shopping,” the process of moving between providers, compromising continuity of care.^15,16^ There is also an inextricable link between anti-fatness and anti-Black racism, as anti-fatness has historically been used to substantiate racial prejudice and today is still used to paint fat Black people as a burden on health care systems.^17^ At best, providers who hold weight bias provide subpar experiences with health care for fat patients and at worst may misdiagnose or prevent patients from seeking necessary care.

This is of particular importance in sexual and reproductive health (SRH) settings, which can be the only touchpoint with health care providers for many patients.^18^ Fat people access preventive services at lower rates, including lifesaving care such as Pap smears and breast cancer screenings,^19^ largely due to real and perceived anti-fat bias by providers.^20^ Patients who are currently or trying to become pregnant are more likely to be shamed or blamed for their size.^15^ Stress, anxiety, resistance to accessing health care, and related factors may be large contributors to the poor health outcomes often attributed to weight alone.^21,22^

Although weight equity is still a developing area of research,^23^ innovations in bias awareness have included provider education and anti-fat bias education modalities, many of which have shown favorable and sustained results.^9,13,23–25^ In-depth bias awareness workshops that include re-education on the myth that individual behavior and choices fully controls body weight and address the socialized link between thinness, morality, and health have been successful.^8,23^ In one 3-hour seminar for clinical trainees, the session significantly lowered participants’ negative attitudes toward fat people.^25^ In another 3-hour conference for medical students that included a small group discussion session, student-reported knowledge and skills regarding weight-inclusive care increased.^9^ Bias reduction and awareness work is growing rapidly and several prominent groups such as the Association of American Medical Colleges and the American Medical Association are creating guidance around weight bias.^26^

The purpose of this program evaluation project was to improve provider weight bias awareness with a workshop series for providers at Planned Parenthood health centers nationwide. Research shows that several sessions of workshops are more effective than single sessions and the most effective workshops employ multiple techniques, including presentations, case studies, and opportunities for exploration and discussion.^9,23–25^ As such, many education modalities were used across the three sessions. The goal of the education is not to lower bias itself, as measuring actual bias is near impossible, but to measure and increase provider bias awareness^27^ and confidence with the Health at Every Size (HAES) principles.^3^

The HAES principles^3^ were created to end weight discrimination and promote access to care regardless of size or weight. The principles include a focus on respectful care through which health care workers acknowledge bias and center equity while promoting healthful movement, intuitive eating, holistic well-being, and enhancing health during the provision of health care rather than focusing on patient weight or BMI.^8,28^ Sessions also framed the ethical implications of weight bias, emphasizing that all people should have access to high-quality care, regardless of their health or weight.^29^ With increased bias awareness, and awareness of the potential harm individual and systemic weight bias has on patient care, providers can provide better care for patients of all sizes.

## Methods

### Participants and logistics

Health care providers from all Planned Parenthood affiliates currently providing care at a Planned Parenthood health center were eligible to attend this multipart workshop. Planned Parenthood is the country’s leading provider of affordable sexual and reproductive health care providing a vital safety net of preventive care by a large, diverse provider group. Potential participants included 1,618 providers, (e.g., nurse practitioners, physicians, midwives, and physician associates) who were sent recruitment emails via a Planned Parenthood clinician listserv. Each potential participant was asked to attend three 1-hour synchronous virtual sessions, which were held weekly for 3 weeks. Participants had the option of attending either a Monday or Friday session each week. Upon registering, participants were provided all dates and times, asked to attest to attending all three workshop sessions, and complete the associated surveys. Reminders were shared 1–2 days prior to each session. Participants created a random participant number, which they input at the beginning of each virtual session to track attendance. Participants received a post-survey immediately after the three-part series, and again 3 months after the final session. Participants were asked to share their unique participant number with each survey to measure participant attrition and pair data for analysis. All surveys were created on Qualtrics and shared over email blasts to participants.

### Curriculum design

The sessions were curated, presented, and facilitated by the lead author along with volunteer experts in weight stigma. The curriculum considered current best practice in anti-bias education, including short readings and presentations consisting of patient stories, the effects of weight stigma, an in-depth look at weight and research in the SRH space, and opportunity for small-group discussion of case studies.^23^

The first workshop outlined the problems with weight stigma, presented evidence on the harms of implicit bias in health care, and highlighted the direct connection between anti-fat bias and anti-Black racism. The second session addressed bias from a provider lens by discussing weight bias-informed approaches to care and presenting flaws in research related to overreliance on weight as a health indicator. It introduced the HAES principles of weight inclusivity, aimed at increasing provider confidence in providing equitable care to fat patients.^3^ The final workshop was an interactive, facilitated, small-group discussion session with theoretical case studies asking participants to practice the learnings from prior sessions with potential real-life clinical situations. Facilitators maintained session notes for thematic analysis. Participants were offered continuing education credits through Duke University as an incentive to complete the entire series and the credits were only awarded if participants attended all sessions.

### Project design

Prior to the series, participants completed an anonymous pre-survey that asked them to self-report their Implicit Association Test (IAT) weight bias score (implicit.harvard.edu). The IAT is a validated measure and prior studies have shown that higher bias scores from providers were associated with fewer follow-ups, shorter length of visits with patients, and that overloaded providers are more likely to exhibit behaviors more strongly influenced by bias.^24,30^ The survey also included modified questions from other validated measures, the Attitudes Toward Obese Persons Scale and Beliefs About Obese Persons Scales (Table 3).^23,24^ Response options are on a 6-point Likert scale wherein lower average scores indicate strong disagreement and higher scores signify strong agreement. To evaluate the project, a pre–post survey design was used, wherein participants completed a survey immediately before the series, directly following the series, and 3 months following the final session. Knowledge, intention, and attitudes related to weight bias in the provision of SRH care and confidence providing unbiased care using the HAES principles were also assessed.^31^ Higher scores on these questions indicate higher confidence levels. Internalization of the HAES principles would be reflected by knowledge scores showing an understanding that higher weight is not a medical condition in and of itself and that fatness is neither good nor bad, but neutral. Given that losing weight and maintaining that weight loss meaningfully is exceedingly challenging, weight-neutral providers should instead be focused on holistic care and health enhancement.^3^ The survey also included an opportunity for open-ended feedback. This project has been formally evaluated using a quality improvement checklist and determined not to be human subjects’ research.

### Data analysis

Using GPower software and the current Planned Parenthood provider population estimate of 1,618, it was calculated that to achieve 95% power with an alpha of 0.05 and a moderate effect size, *N* = 43. Analysis was conducted on complete data, which included participants that attended all three sessions and completed both surveys. To retain matched data for participants, separate paired *t-tests* on SPSS v.28 were used to evaluate the data in pre- and post-survey responses and to compare data from the post-survey with the 3-month follow-up survey. Data for participants who only completed one survey were not used.

## Results

Of the 50 providers that completed all sessions, 30 completed both the pre- and post-surveys and were used in the final analysis. Advanced practice clinicians (APCs) including physician associates, nurse midwives, and nurse practitioners comprised 80% of participants and physicians accounted for the remainder. This is consistent with the national provider distribution at Planned Parenthood, which includes approximately 12% physicians and 87% APCs. Most participants were white women who wear clothing size small or medium and are aged 35–54 years (Table 1). The proportion of the participants self-reporting an IAT score in the category of “little to no automatic preference between fat people and thin people” increased from 23.3% to 50% (*p =* 0.060) (Table 2). Knowledge, attitude and intention, and confidence scores related to weight bias and weight neutral care also improved after workshop participation. The questions focused on the core HAES principles that deemphasize the importance of weight as a measure of health and encourage providers not to pathologize or treat fatness, instead focusing on health promoting behaviors. All statistically significant changes were directionally concordant with the aims of the project (Table 3).

**Table 1.** Participant Demographics

	*n*	*%*				
Which best describes your credentials as a clinician?						
Nurse Midwife	2	6.7%				
Certified Nurse Midwife, Nurse Practitioner	2	6.7%				
Nurse Practitioner	17	56.7%				
PA	3	10.0%				
Physician	6	20.0%				
*What is your age range?*						
25–34	7	23.3%				
35–44	10	33.3%				
45–54	10	33.3%				
55–64	2	6.7%				
65–74	1	3.3%				
*Which best describes your gender identity?*						
Genderqueer/gender fluid, Non-binary	1	3.3%				
Non-binary	2	6.7%				
Woman	27	90.0%				
Man	0	0				
*Which best describes your race/ethnicity? (select all the apply)*						
American Indian or Alaska Native	1	3.3%				
Asian or Asian American	1	3.3%				
Black or African American	1	3.3%				
Hispanic or Latino/a/x,White	1	3.3%				
White	26	86.7%				
*On average, what clothing size do you purchase for yourself?*						
3X	1	3.3%				
XXL/2X	1	3.3%				
XL/1X	4	13.3%				
L	2	6.7%				
M	8	26.7%				
S	12	40.0%				
XS	2	6.7%				

**Table 2.** IAT Results for Pre- and Post-Survey Responses

IAT result	Pre		Post	
	*n*	*%*	*n*	*%*
Little to no automatic preference between fat people and thin people.	7	23.3%	15	50.0
Slight automatic preference for thin people compared with fat people.	7	23.3%	4	13.3
Moderate automatic preference for thin people compared with fat people.	9	30.0%	5	16.7
Strong automatic preference for thin people compared with fat people.	7	23.3%	4	13.3

**Table 3.** Paired Samples t-Test Results for Pre-Versus Post-Survey Responses

	Pre-Test		Post-Test			
Item	*M*	*SD*	*M*	*SD*	*t*	*p*
Intent and attitudes						
I am committed to providing weight loss counseling to fat patients. (*n* = 30)	**3.00**	**1.64**	**2.43**	**1.65**	**2.07**	**0.048**
I think weight loss medications, such as GLP-1 agonists (such as Ozempic and Wegovy) are a good option to talk to my fat patients about.	**3.03**	**1.295**	**2.07**	**1.19**	**6.68**	**<0.001**
Sexual and reproductive health procedures and assessments on fat patients are usually more difficult.	3.55	1.298	3.14	1.217	1.84	0.076
My fat patients tend to be less compliant.	1.69	1.07	1.48	0.82	1.10	0.281
I hold anti-fat bias.	4.34	1.261	3.97	1.349	1.65	0.110
I want to increase my awareness and understanding of my anti-fat bias.	5.86	0.351	5.66	0.670	1.65	0.110
Knowledge						
I understand weight-neutral care and the Health at Every Size principles.	**3.69**	**1.58**	**5.10**	**0.72**	**−4.32**	**<0.001**
It is possible to lose weight and maintain that weight loss.	**4.66**	**1.173**	**3.07**	**1.163**	**6.46**	**<0.001**
Overweight and obesity are diseases and should be treated as such.	**3.28**	**1.68**	**2.24**	**1.24**	**3.98**	**<0.001**
The BMI is a poor indicator of health.	5.07	1.033	5.41	1.268	−1.24	0.224
Sexual and reproductive health procedures on fat patients are usually riskier.	**2.45**	**0.98**	**1.93**	**0.92**	**2.64**	**0.013**
Weight bias in providers contributes to poor health in fat patients.	5.24	1.09	5.59	0.68	−1.58	0.125
Confidence						
I feel confident providing weight-neutral care and using the Health at Every Size principles in my clinical practice.	**3.28**	**1.46**	**4.72**	**0.79**	**−5.19**	**<0.001**
I feel confident in my knowledge of medication efficacy for contraception methods and emergency contraception for fat patients.	4.83	1.25	5.03	1.08	−0.812	0.424
I feel confident in my understanding of how anti-fatness and anti-Blackness in health care intersect.	**4.17**	**1.28**	**5.10**	**0.77**	**−4.31**	**<0.001**
I feel confident about how to speak to fat patients respectfully	**4.62**	**0.820**	**5.17**	**0.889**	**−3.13**	**0.004**
I feel confident advocating for procedure and policy changes at my organization that improve the care and experience of fat people.	**3.90**	**1.472**	**4.69**	**1.004**	**−3.46**	**0.002**
I feel confident knowing when I do and don’t need to factor weight into a patient’s care.	**4.10**	**1.175**	**4.97**	**0.944**	**−3.04**	**0.005**

Bolded items show significant changes from pre- to post-education based on the level of significance of 0.05. Mean scores are in the range of 1–6 based on participant response on a Likert scale, wherein 1 = I strongly disagree, 2 = I moderately disagree, 3 = I slightly disagree, 4 = I slightly agree, 5 = I moderately agree, and 6 = I strongly agree.

Some of the responses in the survey that did not show significant changes between the pre- and post-survey were questions around intentions or knowledge, such as whether BMI is a good indicator of health or if the participant has a desire to increase their understanding and awareness of their anti-fat bias (Table 3). Participants (*n* = 15) completing both the post-survey and the 3-month follow-up survey showed a statistically significant retention of improvement in confidence in speaking respectfully to fat patients (*p* = 0.029).

Several themes emerged from the participant discussion during the third session of the workshops*.* Participants displayed a meaningful understanding of the need to challenge bias at a systems level and an earnest shift away from weight-centered approaches to care. Across all small group discussion sessions, participants verbalized a commitment to continue learning and confronting their biases and a deep recognition of the intersectionality between weight bias and racism. One participant shared that after this education, they will “always re-center with the question ‘how would I approach this visit with a patient who was in a smaller body?’” Another noted that this workshop “made me question so much of my medical training and what is actually backed by science versus conflated with bias.” Many expressed feeling energized to engage in an educational and advocacy capacity on issues related to weight stigma. Several participants agreed that a weight-focused care paradigm in medicine led to, as one participant phrased it, “a chain reaction of non-evidence based and bias care.”

## Discussion

This program achieved its aim of increasing SRH provider awareness of their biases related to weight and increasing their confidence providing care using the HAES principles. Health care providers with patient-centered communication skills, empathy, and awareness of their biases (with a plan to mitigate that bias) are an important part of improving health outcomes in fat patients. This project provides critical insights for improving weight bias awareness in the population of practicing SRH clinicians rather than students, the target population in most prior interventions.^8–10,24–26^ Although improvements in participant IAT scores did not reach statistical significance, they represent a clinically significant shift away from an automatic bias against fat patients toward an awareness to be intentional about recognizing bias and not acting on it. The IAT is a widely used tool to measure the existence of weight bias in provider populations, but it is not commonly used to measure changes in that bias after an intervention, as in this project.^30^

Participants in this project demonstrated an improvement in awareness and knowledge of weight bias after bias awareness training, as in prior studies.^8,13,23–25^ However, this project uniquely employed a series of in-depth workshops over several sessions, which adds to the body of work demonstrating that longer form learning designs allow for the gradual accrual of knowledge and the space to practice analysis, evaluation, and application of that knowledge.^9,32^ Having multiple sessions may improve the breadth and depth of content participants can internalize, and having time between sessions to reflect on the material is beneficial to learning and retaining new information.

The majority of participants entered the training strongly agreeing that they want to increase their awareness and understanding of their anti-fat bias, indicating a readiness for education focused on advocacy, analysis, and behavior change.^32^ Prior to the education, most participants also had a strong baseline understanding that the BMI is a poor indicator of health but limited awareness of HAES principles and their applications. This is important to consider when designing future interventions and demonstrates how pre-test responses can inform not only education design but also appropriate measures of success.

During the small group discussion sessions, participants demonstrated the skills and ideas learned during the initial two sessions and a desire to go beyond the workshops to support their fat patients. Participants also shared systematic and interpersonal barriers for providing unbiased weight care. For instance, some electronic medical records may hinder unbiased care because of many preset alerts around weight. There is a need for qualitative research to explore the themes of these systemic barriers and advocacy and policy change beyond the individual-level that incorporates removing built-in biases from informatics systems.

Participants not only displayed an increased confidence in their understanding of weight-neutral and respectful care, but an increased intent to act on this knowledge. In an open-ended response question in the post-survey, participants shared a deep appreciation for the sessions and the knowledge and skills they provided them with to help mitigate their weight bias. Several participants went on to describe the ways they plan to continue their learning, such as reading suggested literature, and one shared they had already begun planning to share weight-bias information with all staff at their health center, including bringing in an expert presenter. One participant said they changed a patient’s plan of care because of the workshop. This feedback indicates that the project increased bias awareness as well as participant understanding that bias mitigation is an ongoing process.

Our findings should be interpreted within the context of this project. This project used a convenience sample of SRH providers, was limited to 4 months, and did not measure provider behavior. Because of the voluntary nature of participation in this project, there is inherent bias in the results, as participants likely had more interest and concern about weight bias than the average provider. A required training may yield more significant results as participants would start with higher bias and lower knowledge, which could result in participants being less influenceable. Future work should include longitudinal analysis of participants to measure knowledge retention and behavior change. Despite efforts to avoid attrition and low response rates, the project experienced both. Future projects might improve this by shortening surveys, offering more incentives for survey completion, or not offering CE credit until the completion of all surveys. Further, although providers were the population of focus in this limited-scope project, as most evidence on the detrimental effects of bias in health care focuses on provider bias, future work must include all staff that interacts with patients. In addition, no patient data were collected. Future studies should incorporate patients’ experiences on the direct impact of increased weight bias awareness on patient care, such as monitoring trends in patient health and care utilization, patient–provider communication, and patient experience.

Because of the national scope of the population, this project was conducted via a live webinar. In-person interaction may facilitate stronger discussions and more focused engagement by participants. The design of this project inherently suffers from self-selection bias as those most interested or informed are the most likely to participate. To combat this in future projects, institutions could require weight bias education for all providers or employees.

### Health equity implications

This project extends current literature supporting the value in a multi-step model of clinician education to combat provider weight bias in a meaningful way. While health equity guides the clinical practice of many organizations, size equity has yet to be meaningfully addressed on a universal scale. Given the inextricable link between anti-fatness and racism, addressing weight bias can be a meaningfully integrated part of organizations’ work on combating racism in health care.^17^ Participants in anti-racism workshops display an increased awareness of not only racial bias but other kinds of bias as well.^33^ Therefore, weight bias programming could have a potentially symbiotic relationship with other health equity and anti-racism initiatives.

Because of Planned Parenthood’s influence on SRH, changes made inside the organization could act as a catalyst for weight equity in the wider SRH community. Providers armed with the knowledge of the harms of weight bias are more likely to provide more inclusive, patient-centered care that focuses less on weight and BMI and more on health-improving behaviors and total wellbeing. In addition, providers educated in bias are more likely to act as changemakers for policies in their clinics as well as advocate for changes in the general health care landscape. Health policies addressing racial bias in terms of BMI are crucial to ensuring equity in health care. All patients deserve care that is free of discrimination, including weight-based discrimination.

### IRB waiver statement

This project has been formally evaluated using a quality improvement checklist and determined not to be human subjects’ research.

## Notes

### Abbreviations Used

APC: advanced practice clinician
BMI: body mass index
HAES: Health at Every Size
IAT: Implicit Association Test
SRH: sexual and reproductive health
